# Visceral Adipose Tissue Indices Independently Correlated with Obstructive Sleep Apnea in Patients with Type 2 Diabetes

**DOI:** 10.1155/2022/4950528

**Published:** 2022-02-10

**Authors:** Caiyu Zheng, Xuanling Zheng, Xiaoyan Lin, Jiawen Ye, Ziqing Xu, Huixian Hu, Wengui Wang, Caoxin Huang, Jianqing Tian, Changqin Liu

**Affiliations:** ^1^Department of Endocrinology, Fujian Medical University Xiamen Humanity Hospital, Xiamen, Fujian Province, China; ^2^School of Medicine, Xiamen University, Xiamen, Fujian Province, China; ^3^Department of Endocrinology and Diabetes, The First Affiliated Hospital of Xiamen University, School of Medicine, Xiamen University, Xiamen, Fujian Province, China; ^4^The Third Clinical Medical College, Fujian Medical University, Fuzhou, Fujian Province, China; ^5^Fujian Province Key Laboratory of Diabetes Translational Medicine, Xiamen, Fujian Province, China; ^6^Xiamen Diabetes Institute, The First Affiliated Hospital of Xiamen University, School of Medicine, Xiamen University, Xiamen, Fujian Province, China; ^7^Xiamen Diabetes Prevention and Control Center, Xiamen, Fujian Province, China

## Abstract

**Aims:**

We aimed to explore whether visceral adiposity indices were significantly associated with obstructive sleep apnea (OSA) in type 2 diabetes (T2DM) patients.

**Methods:**

100 patients with T2DM who underwent overnight polysomnography were analyzed in this study. Anthropometric data, lipid profiles, and glycemic parameters were recorded. Body fat percentage (BFP) and visceral adipose tissue area (VAT area) were collected from a whole body scan using dual-energy X-ray absorptiometry (DXA). Multivariate logistic regression analysis was performed to explore the associations of AHI with BFP, VAT area, and CVAI.

**Results:**

The prevalence rate of OSA was 80%, and the mean (±SD) of age was 47.0 ± 13.6 years. Apnea-hypopnea index (AHI) was significantly and positively associated with either VAT area (*r* = 0.433, *p* ≤ 0.001) or Chinese visceral adiposity index (CVAI) (*r* = 0.355, *p* ≤ 0.001) but not for BFP (*r* = 0.107, *p* = 0.294). Multivariate logistic regression analyses showed that VAT area and CVAI were significantly associated with increased risk of OSA, and the adjusted ORs were (95% CI) 1.025 (1.003-1.047, *p* = 0.023) and 1.018 (1.002-1.034, *p* = 0.030), respectively. However, there was no significant association between BFP and increased risk of OSA.

**Conclusions:**

VAT area and CVAI were independent risk factors of OSA in the patients with T2DM.

## 1. Introduction

Obstructive sleep apnea (OSA) is a disease characterized by recurrent episodes of shallow or paused breathing during sleep, which will lead to intermittent hypoxia (IH) and sleep disruption due to partial or complete obstructions of the upper airway. Type 2 diabetes (T2DM) is a serious chronic disease which brings about a series of complications such as hypertension and OSA. There are close correlations among OSA, T2DM, and obesity. Approximately 48% diabetic patients had an apnea − hypopnea index (AHI) > 10/h [1]. OSA can lead to an increase in insulin resistance, systemic inflammation, and sympathetic tone, which underlie the development and maintenance of diabetes [2]. Previous studies have revealed that progression from mild OSA to severe OSA led to increased frequency of abnormal glucose metabolism [3]. Also, increasing evidences demonstrated that obesity could lead to the occurrence of OSA and T2DM [4].

Currently, anthropometric measures such as the body mass index (BMI), waist circumference (WC), and waist-hip ratio (WHR) were widely used to assess obesity. However, indirect measures such as BMI failed to distinguish between fat, muscle, and bone mass and lead to misclassification of those with excess body fat [5]. A large of studies suggested that the adipose tissue was an important endocrine organ [6]. A number of cytokines and inflammatory factors secreted by the adipose tissue regulated the homeostasis of glucose and lipid metabolism and played key roles in the morbidity of OSA and diabetes [7]. Further, visceral adipose tissue with excess visceral fat having a more serious influence was thought to differentially contribute to disease risk and found to be associated with increased risk of T2DM in those with OSA [8].

Dual-energy X-ray absorptiometry (DXA) has been considered the gold standard for measuring visceral fat [9]. Estimation of VAT area by DXA is a newer technique with shorter scanning time, less radiation exposure, and lower cost [10]. Previous studies had indicated that VAT area was associated with the metabolic syndrome [11]. Besides, a Chinese visceral adiposity index (CVAI), based on BMI, age, total triglycerides (TG), WC, and high-density lipoprotein cholesterol (HDL-c), was established to estimate visceral adiposity and predict the incidence of metabolic disorders, and several studies demonstrated that CVAI was associated with T2DM [12]. Xia et al. [13] found that the visceral adiposity estimated by CVAI was superior to the traditional estimates of obesity for the prediction of incident prediabetes and diabetes in Chinese adults. However, among the individuals with T2DM, the relationship of VAT area, CVAI, and OSA remained unclear. So, the current study was aimed to explore whether VAT area and CVAI were significantly associated with OSA in T2DM patients.

## 2. Materials and Methods

### 2.1. Participants

A total of 200 adult subjects with T2DM were screened in the study from the First Affiliated Hospital, Xiamen University, China, from September 2017 to January 2019. The subjects admitted routine check-up evaluations and underwent overnight polysomnography (PSG). Whole body DXA measurements of body fat percentage were performed in all patients. Exclusion criteria had been described in detail as before [14]. 21 subjects were excluded because the sleep was less than 4 hours at night. 39 Subjects were excluded because of the deficiency of body fat percentage. 40 patients without questionnaire data were further excluded from the study. Finally, 100 patients (70 men and 30 women) were left for analyses. The study was approved by the Human Research Ethics Committee of the Xiamen First Hospital. All subjects provided written informed consent.

### 2.2. Anthropometric and Biochemical Measurements

Anthropometric measurements including body weight, height, WC, blood pressure (BP), and BMI were described previously [15, 16]. BMI was calculated as the weight in kilograms divided by the square of the height in meters. After 12-hour fasting, all blood samples were obtained. Blood samples were measured in the central laboratory of the First Affiliated Hospital, Xiamen University. Briefly, hemoglobin A1c (HbA1c) was measured by the Bio-Rad Variant Hemoglobin A1c assay. TG, total cholesterol (TC), high-density lipoprotein cholesterol (HDL-c), and low-density lipoprotein cholesterol (LDL-c) were determined on a HITACHI 7450 analyzer (HITACHI, Tokyo, Japan).

### 2.3. Polysomnography

Polysomnography (PSG) is the gold standard method for diagnosing and assessing the severity of OSA [16]. All subjects underwent an overnight PSG from 11 : 00 pm to 7 : 00 am according to the standard techniques with monitoring the electroencephalogram (EEG), electrooculogram (EOG), electromyogram (EMG), flow, thoracic and abdominal respiratory effort, oximetry, and body position. Polysomnographic recordings were interpreted in accordance with the current American Academy of Sleep Medicine (AASM) guidelines [17]. The monitoring was repeated on a second night if subjective sleep latency exceeded 2 hours on the first night. AHI was defined as the total number of obstructive apnea and hypopnea per hour of sleep, and the severity of OSA was determined by AHI. OSA is defined based on thresholds: OSA is an AHI ≥ 5/h [16].

### 2.4. Dual-Energy X-Ray Absorptiometry

DXA offers a low radiation alternative and has been used to measure total and regional body composition for many years. In our study, whole body DXA measurement of body composition was performed in all patients using a Hologic Discovery A (Hologic Inc., Bedford, MA). The patients underwent the scan with minimal clothing and not wearing any metal object. In addition, they were asked to stay as calm and quiet as possible during the scan time. Once the DXA scan was performed, we calculated the data of body fat percentage (BFP) and VAT area.

### 2.5. Chinese CVAI

Based on the prediction equation for visceral adiposity in Chinese populations, CVAI was used to estimate the visceral adiposity area [18]. CVAI (male) = −267.93 + 0.68∗age + 0.03∗BMI + 4.00∗WC + 22.00∗Log10 TG − 16.32∗HDL − c. CVAI (female) = −187.32 + 1.71∗age + 4.23∗BMI + 1.12∗WC + 39.76∗Log10 TG − 11.66∗HDL − c.

### 2.6. Statistical Analysis

Data were analyzed with the use of IBM SPSS Statistics 21.0. The results are expressed as mean ± standard deviation (SD) or median (interquartile ranges) for continuous. Differences between groups were analyzed using Mann-Whitney *U* test or ANOVA for continuous variables and chi-square test for categorical variables. The correlation of AHI with BFP, VAT area, and CVAI was analyzed using Pearson's correlation analysis. Multivariable logistic regression analysis was used to calculate the adjusted odds ratios (OR) and 95% confidence interval (CI) of BFP, VAT area, and CVAI for OSA in different models with adjustment for potential confounders. No confounders were adjusted in model 1; age, gender, T2DM duration, regular drinking, current smoking, and hypertension were adjusted in model 2; For both BFP and VAT area, BMI, HbA1c, TG, LDL-c, and HDL-c were further adjusted for in model 3, but for CVAI, only HbA1c and LDL-c were further adjusted for in model 3. All *p* values presented are two-tailed, and values less than 0.05 are considered statistically significant.

## 3. Results

Totally, there were 100 diabetic patients left for the present study, and among them, 70 (70%) were men and 30 (30%) were women. The overall prevalence rate of OSA was 80%, and the mean (±SD) of age was 47.0 ± 13.6 years in the current small cohort.

### 3.1. Clinical Characteristics Categorized by AHI

**Figure 1:**
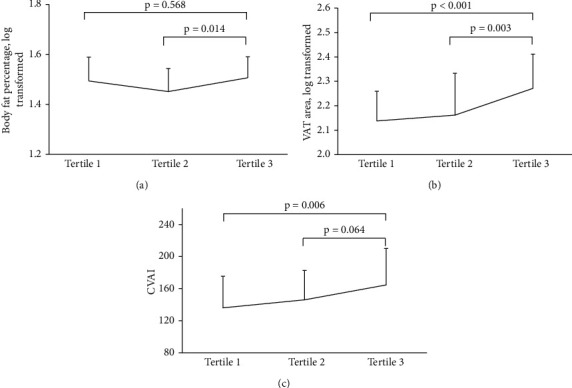
Distributions of body fat percentage, VAT area (log-transformed), and CVAI stratified by AHI tertiles.

Table 1 shows the differences of demographic and clinical characteristics categorized by AHI (tertile 1, tertile 2, and tertile3). There were significant differences in the Epworth sleepiness score (ESS) (2.0, 6.0, and 10.0, respectively, *p* < 0.001), HDL-c (1.07, 1.08, and 1.24, respectively, *p* < 0.05), LDL-c (2.83 ± 1.02, 2.53 ± 0.85, and 3.15 ± 0.84, respectively, *p* < 0.05), BFP (31.5, 27.4, and 30.8, respectively, *p* < 0.05), CVAI (136.1 ± 39.4, 146.0 ± 36.6, and 164.6 ± 45.4, respectively, *p* < 0.001), and VAT area (124.0, 138.5, and 180.0, respectively, *p* < 0.001) among three groups. But, there were no significant differences among these three groups in age, regular drinking, current smoking, T2DM duration (*p* = 0.421), SBP (133.8 ± 16.0, 131.1 ± 14.7, and 131.2 ± 14.5, respectively, *p* = 0.721), DBP (83.6 ± 9.9, 84.3 ± 9.4, and 83.0 ± 8.6, respectively, *p* = 0.852), BMI (28.46 ± 4.02, 28.60 ± 5.39, and 30.75 ± 4.79, respectively, *p* > 0.05), WC (98.4 ± 11.7, 99.5 ± 10.8, and 104.9 ± 13.1, respectively, *p* > 0.05), HC (102.7 ± 8.8, 101.3 ± 8.4, and 105.2 ± 13.2, respectively, *p* > 0.05), WHR (0.96 ± 0.07, 0.98 ± 0.08, and 1.00 ± 0.08, respectively, *p* > 0.05).  Figure 1 showed the distributions of BFP, VAT area (log-transformed), and CVAI stratified by AHI tertiles. As for BFP, there was no significant difference in the comparison between tertile 1 and tertile 3.

### 3.2. Associations of VAT Area, CVAI, and Body Fat Percentage with OSA

**Figure 2:**
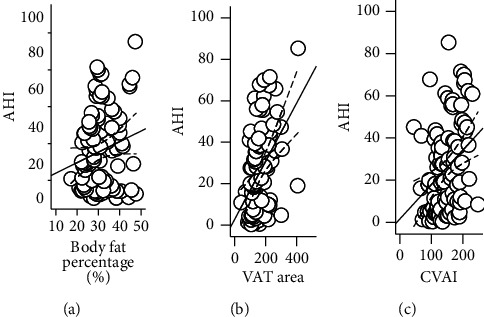
The correlation of AHI with body fat percentage, VAT area, and CVAI.

Furthermore, the correlation of AHI with BFP, VAT area, and CVAI was analyzed using Pearson's correlation analysis in Figure 2. There were significant and positive associations between VAT area (*r* = 0.433, *p* = <0.001) and CVAI (*r* = 0.355, *p* = <0.001) with AHI. But the positive correlation between BFP and AHI (*r* = 0.107, *p* = 0.294) was not statistically significant.

Table 2 shows the association of VAT area, CVAI, and BFP with OSA in patients with T2DM using multivariable logistic regression. As for VAT area, increased VAT area was significantly associated with increased risk of OSA, and the OR (95% CI) was 1.011 (1.000-1.023, *p* = 0.047) in model 1 without any confounding factors. In model 2 with adjustment of age, gender, T2DM duration, regular drinking, current smoking, and hypertension, VAT area was still significantly associated with increased risk of OSA with the adjusted OR (95% CI) of 1.017 (1.005-1.030, *p* = 0.006). In model 3, VAT area was still significantly associated with increased risk of OSA with the adjusted OR (95% CI) of 1.025 (1.003-1.047, *p* = 0.023) after further adjusting for BMI, HbA1c, TG, LDL-c, and HDL-c. However, as for BFP, there were no significant associations between BFP and increased risk of OSA in model 1, model 2, and model 3 with the same adjustment in VAT area. As for CVAI, increased CVAI was significantly associated with increased risk of OSA in model 1 and model 2, and the OR (95% CI) were 1.013 (1.000-1.026, *p* = 0.044) and 1.015 (1.001-1.030, *p* = 0.040), respectively. In model 3, which was further adjusted for HbA1c and LDL-c, CVAI was still significantly associated with OSA with the adjusted OR (95% CI) of 1.018 (1.002-1.034, *p* = 0.030).

## 4. Discussion

In the present study, we found that there were significant differences in HDL-c, LDL-c, BFP, CVAI, and VAT area among the tertiles of AHI, and Pearson's correlation analysis showed that the VAT area and CVAI were significantly and positively associated with AHI. Furthermore, multivariable logistic regression analysis showed that VAT area and CVAI were the independent risk factors of OSA in patients with T2DM with adjustment for potential confounding factors.

Over the past few decades, a large body of evidence had identified the associations between OSA and T2DM. OSA as a significant contributing factor to impaired glucose metabolism appeared well proven [19]. In the Sleep Heart Health Study, OSA was independently associated with fasting glucose intolerance and the study also showed that glycemic control worsened with more severe OSA [20]. Several epidemiological studies also identified that OSA was an independent risk factor for the development of T2DM [21]. After adjusting for age, sex, BMI, and WC, OSA was associated with occult diabetes, impaired fasting glucose (IFG), and IFG plus impaired glucose tolerance (IGT) [22].

Also, there was also a close relationship between obesity and OSA. Obesity is the strongest risk factor for the development of OSA, and a large epidemiological study had found that there was a strong association between weight gain and increased risk of OSA [23]. Simple anthropometric measures such as BMI and WC were used to assess obesity in common. However, previous studies showed that visceral adiposity may be superior to anthropometric indices of obesity (such as BMI or WC) to identify diabetes and cardiovascular disease and was recognized as an important risk factor for cardiometabolic disease [24]. Moreover, OSA patients were found to have more visceral adiposity than BMI-matched obese controls, and visceral adiposity had been closely associated with insulin resistance with increased lipolysis [25]. A Spearman correlation analysis illustrated that VAT area was significantly correlated with AHI, mean pulse oxygen saturation (SpO_2_), and metabolic indicators [26]. Besides, CVAI was also an applicable and reliable index for evaluation of visceral fat dysfunction, which was strongly and positively associated with visceral fat area [27]. Han et al. found that CVAI was the best performance in predicting incident T2DM and it might be an applicable and reliable indicator to identify people at high risk of T2DM. In our study, we also found that CVAI was an independent risk factor of OSA. The mechanisms are complex and not fully understood, in which inflammation may be one of the possible mechanisms. Increases in T cell activation and leukocyte-endothelial cellular interactions are linked to vascular inflammation and remodeling [28]. Moreover, a large study in adolescents found that visceral fat was most significantly associated with OSA comparing with other types of adipose tissue, which indicated that inflammation was the significant mediating link in the relationship between OSA and visceral adiposity [29]. During this process, T2DM may contribute to chronic inflammation, and the inflammation could further lead to the occurrence of OSA. The inflammation originating from obese adipose tissue develops systemically and contributes to hyperglycemia. Hyperglycemia can also contribute to chronic, low-grade inflammation resulting in compromised immune function [30]. The release of inflammatory mediators is prompted by hyperglycemia and mediated by oxidative stress confirming the link between inflammation, oxidative stress, and diabetes mellitus [31].

Our study also had a few limitations. First, the causal relationship between body fat distribution and OSA could not be determined because of the cross-sectional study design. Second, the present subjects were not randomly sampled, and the selection bias was obviously. Third, the number of patients in this study was relatively small; therefore, our results should be confirmed in the future prospective cohort studies with larger sample size.

## 5. Conclusion

VAT area and CVAI were the independent risk factors of OSA in patients with T2DM and should be considered in OSA management in clinical.

## Figures and Tables

**Table 1:** Characteristics of subjects by tertiles of AHI in patients with T2DM.

	Tertile 1	Tertile 2	Tertile 3	Total	*p* value
*n* (%)	32	35	33	100	
Age (years)	44.2 ± 14.0	49.5 ± 13.5	47.2 ± 13.1	47.0 ± 13.6	0.285
Gender (*n*, %)					0.119
Woman	14 (14%)	8 (8%)	8 (8.0%)	30	
Man	18 (18.0%)	27 (27%)	25 (25%)	70	
Regular drinking (*n*, %)	12 (12%)	20 (20%)	12 (12%)	44	0.151
Current smoking (*n*, %)	9(9%)	15(15%)	9(9%)	33	0.305
ESS	2.0 (2.0-4.0)	6.0 (2.0-10.0)	10.0 (5.5-12.0)	6.0 (2.01-10.0)	<0.001#
DM duration	4.0 (1.0-11.0)	6.0 (1.0-10.0)	4.5 (1.0-8.8)	5.0 (1.0-10.0)	0.421
SBP (mmHg)	133.8 ± 16.0	131.1 ± 14.7	131.2 ± 14.5	132.0 ± 15.0	0.721
DBP (mmHg)	83.6 ± 9.9	84.3 ± 9.4	83.0 ± 8.6	83.6 ± 9.2	0.852
BMI (kg/m^2^)	28.46 ± 4.02	28.60 ± 5.39	30.75 ± 4.79	29.26 ± 4.86	0.098
Waist circumference (cm)	98.4 ± 11.7	99.5 ± 10.8	104.9 ± 13.1	101.0 ± 12.1	0.064
Hip circumference (cm)	102.7 ± 8.8	101.3 ± 8.4	105.2 ± 13.2	103.0 ± 10.4	0.290
WHR	0.96 ± 0.07	0.98 ± 0.08	1.00 ± 0.08	0.98 ± 0.08	0.098
HbA1c (%)	9.10 ± 1.80	9.85 ± 2.05	9.72 ± 2.39	9.57 ± 2.10	0.308
TG (mmol/L)	1.86 (1.39-2.59)	1.63 (1.22-2.95)	2.03 (1.39-3.39)	1.92 (1.27-2.94)	0.528
TC (mmol/L)	4.91 ± 1.41	4.89 ± 1.70	5.51 ± 1.09	5.10 ± 1.44	0.136
HDL-c (mmol/L)	1.07 (0.96-1.21)	1.08 (0.97-1.38)	1.24 (1.08-1.40)	1.12 (1.01-1.35)	0.042^∗^
LDL-c (mmol/L)	2.83 ± 1.02	2.53 ± 0.85	3.15 ± 0.84	2.83 ± 0.93	0.021^∗^
BFP (%)	31.5 (26.2-36.8)	27.4 (24.5-31.1)	30.8 (27.9-36.9)	29.8 (26.1-36.2)	0.028^∗^
VAT area (cm^2^)	124.0 (113.0-165.0)	138.5 (121.5-191.5)	180.0 (150.5-229.5)	155.0 (123.5-192.3)	<0.001#
CVAI	136.1 ± 39.4	146.0 ± 36.6	164.6 ± 45.4	149.0 ± 41.9	0.019
AHI	4.4 (2.8-6.8)	19.5 (15.6-27.6)	47.3 (39.7-61.1)	19.8 (7.0-39.9)	<0.001#

^∗^ *p* < 0.05; ^#^*p* < 0.001. Data were presented as mean ± SD or median (interquartile ranges) for continuous variables and numbers (proportions) for categorical variables. *Abbreviations*: T2DM: type 2 diabetes mellitus; AHI: apnea-hypopnea index; ESS: Epworth sleepiness scale; DM: diabetes mellitus; SBP: systolic pressure; DBP: diastolic pressure; BMI: body mass index; WHR: waist-to-hip ratio; HbA1c: hemoglobin A1c; TG: triglycerides; TC: total cholesterol; HDL-c: high-density lipoprotein cholesterol; LDL-c: low-density lipoprotein cholesterol; BFP: body fat percentage; BFP: body fat percentage; VAT: visceral adipose tissue; CVAI: Chinese visceral adiposity index.

**Table 2:** Associations of VAT area, CVAI, and body fat percentage with OSA in patients with T2DM.

	Logistic regression on OSA		
	ORs	95% CI	*p* value
BFP			
Model 1	0.942	0.878-1.011	0.097
Model 2	0.998	0.884-1.126	0.973
Model 3	0.859	0.714-1.033	0.106
VAT area			
Model 1	1.011	1.000-1.023	0.047∗
Model 2	1.017	1.005-1.030	0.006∗
Model 3	1.025	1.003-1.047	0.023∗
CVAI			
Model 1	1.013	1.000-1.026	0.044∗
Model 2	1.015	1.001-1.030	0.040∗
Model 3	1.018	1.002-1.034	0.030∗

^∗^ *p* < 0.05; ^#^*p* < 0.001. AHI was log-transformed. *For BFP and VAT area*, model 1 without adjustment and model 2 was further adjusted for gender, T2DM duration, regular drinking, current smoking, and hypertension. *Model 3* was further adjusted for BMI, HbA1c, TG, LDL-c, and HDL-c. *For CVAI*, model 1 without adjustment and model 2 was further adjusted for gender, T2DM duration, regular drinking, current smoking, and hypertension. *Model 3* was further adjusted for HbA1c and LDL-c. *Abbreviations:* T2DM: type 2 diabetes mellitus; OSA: obstructive sleep apnea syndrome; DM: diabetes mellitus; BMI: body mass index; WHR: waist-to-hip ratio; HbA1c: hemoglobin A1c; TG: triglycerides; TC: total cholesterol; HDL-c: high-density lipoprotein cholesterol; LDL-c: low-density lipoprotein cholesterol; BFP: body fat percentage; BFP: body fat percentage; VAT: visceral adipose tissue; CVAI: Chinese visceral adiposity index; AHI: apnea-hypopnea index.

## Data Availability

The datasets used and/or analyzed during the present study are available from the corresponding author upon reasonable request.
